# Domain-swapped cytochrome *cb*_562_ dimer and its nanocage encapsulating a Zn–SO_4_ cluster in the internal cavity†
†Electronic supplementary information (ESI) available: Experimental procedure, size exclusion chromatogram, DSC thermogram, protein structure and statistics of data collection. See DOI: 10.1039/c5sc02428e


**DOI:** 10.1039/c5sc02428e

**Published:** 2015-09-22

**Authors:** Takaaki Miyamoto, Mai Kuribayashi, Satoshi Nagao, Yasuhito Shomura, Yoshiki Higuchi, Shun Hirota

**Affiliations:** a Graduate School of Materials Science , Nara Institute of Science and Technology , 8916-5 Takayama, Ikoma , Nara 630-0192 , Japan . Email: hirota@ms.naist.jp; b Graduate School of Science and Engineering , Ibaraki University , 4-12-1, Nakanarusawa , Hitachi , Ibaraki 316-8511 , Japan; c Department of Life Science , Graduate School of Life Science , University of Hyogo , 3-2-1 Koto, Kamigori-cho, Ako-gun , Hyogo 678-1297 , Japan; d RIKEN SPring-8 Center , 1-1-1 Koto, Sayo-cho, Sayo-gun , Hyogo 679-5148 , Japan

## Abstract

Protein nanostructures have been gaining in interest, along with developments in new methods for construction of novel nanostructures. We have previously shown that *c*-type cytochromes and myoglobin form oligomers by domain swapping. Herein, we show that a four-helix bundle protein cyt *cb*_562_, with the cyt *b*_562_ heme attached to the protein moiety by two Cys residues insertion, forms a domain-swapped dimer. Dimeric cyt *cb*_562_ did not dissociate to monomers at 4 °C, whereas dimeric cyt *b*_562_ dissociated under the same conditions, showing that heme attachment to the protein moiety stabilizes the domain-swapped structure. According to X-ray crystallographic analysis of dimeric cyt *cb*_562_, the two helices in the N-terminal region of one protomer interacted with the other two helices in the C-terminal region of the other protomer, where Lys51–Asp54 served as a hinge loop. The heme coordination structure of the dimer was similar to that of the monomer. In the crystal, three domain-swapped cyt *cb*_562_ dimers formed a unique cage structure with a Zn–SO_4_ cluster inside the cavity. The Zn–SO_4_ cluster consisted of fifteen Zn^2+^ and seven SO_4_^2–^ ions, whereas six additional Zn^2+^ ions were detected inside the cavity. The cage structure was stabilized by coordination of the amino acid side chains of the dimers to the Zn^2+^ ions and connection of two four-helix bundle units through the conformation-adjustable hinge loop. These results show that domain swapping can be applied in the construction of unique protein nanostructures.

## Abstract 2

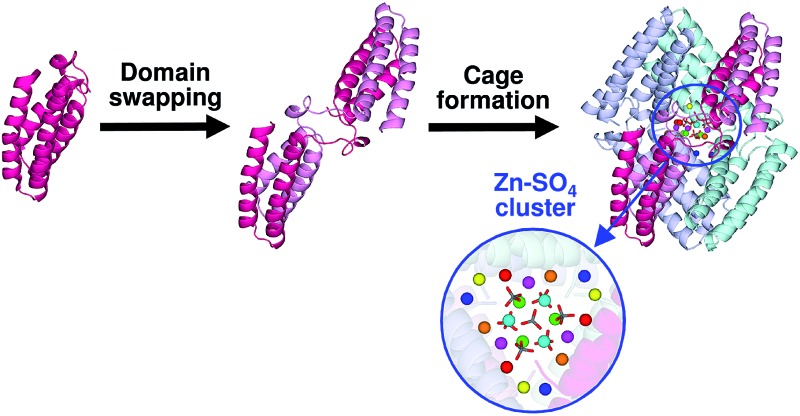
Three domain-swapped cytochrome *cb*_562_ dimers formed a unique cage structure with a Zn–SO_4_ cluster inside the cavity.

## Introduction

Protein nanostructures for development of artificial biomaterials have been constructed under various methods, such as chemical modification,^1^ disulfide bonding,^2^ cross-linking,^3^ computational design,^4^ carbon nanotube templating,^5^ metal coordination,^6^ protein–peptide tag interaction,^7^ and protein fusion.^8^ However, increase in protein nanostructure variety is desirable and therefore development of other methods such as domain swapping is necessary. Domain swapping has attracted attention as a mechanism of protein oligomerization, and reports on domain swapping have been increasing.^9^ In domain swapping, a secondary structural region or a domain of one protein molecule is replaced with the corresponding region or domain of another protein molecule.^10^ We have previously shown that small spherical heme proteins, *c*-type cytochrome (cyt) proteins and horse myoglobin (Mb), form oligomers by domain swapping.^11^ In the dimer and trimer of horse cyt *c*, the C-terminal α-helix domain swapped with the corresponding region of other molecules. In dimeric *Pseudomonas aeruginosa* (PA) cyt *c*_551_ and dimeric *Hydrogenobacter thermophilus* (HT) cyt *c*_552_, the region containing the N-terminal α-helix and heme was swapped.^11b,d^ Previously, we proposed that the unstable loop region has a tendency to become a hinge loop for domain swapping in cyt *c* family proteins.^11*d*^ In domain-swapped dimeric horse Mb, each active site consisted of two different protomers, and new long α-helices were formed by the E and F helices and the EF-loop of the original monomer.^11*c*^ Although apoMb formed a dimer, a large amount of apoMb dissociated to monomers by incubation at 37 °C for 30 min, whereas holoMb oligomers did not dissociate under the same conditions.^11*c*^ These results indicated that the Mb dimer was stabilized by heme binding to the protein.

Cyt *b*_562_ from *E. coli* is a relatively small (MW: ∼12 000) heme protein responsible for electron transfer in the periplasm.^12^ Owing to its simple four-helix bundle structure, cyt *b*_562_ is frequently used as a model for studying protein stability and folding.^13^ Cyt *b*_562_ from *E. coli* is composed of 106 amino acids, and four α-helices are arranged in an antiparallel orientation.^14^ Met7 and His102 are coordinated to the heme iron in cyt *b*_562_, where the heme may dissociate from the protein moiety. A *c*-type cyt *b*_562_ (cyt *cb*_562_) has been constructed by Barker and co-workers by replacing Arg98 and Tyr101 with Cys residues and introducing two covalent thioether bonds between the heme and protein moiety.^15^ Gray and co-workers have shown that the covalent attachment of the heme to the protein moiety in cyt *cb*_562_ increases its protein stability without perturbation in the structure and folding process from those of the wild-type protein (cyt *b*_562_).^16^

Tezcan and co-workers have constructed cyt *cb*_562_ nanostructures by introducing intermolecular interaction sites at the protein surface.^6b,17^ A cyt *cb*_562_ variant with two metal-chelating bis-His motifs at its surface has been shown to assemble into a tetramer, trimer and dimer through Zn^2+^, Ni^2+^ and Cu^2+^ coordination, respectively.^17*c*^ Cage-like tetrahedral dodecamers have been constructed in the crystal lattice of another cyt *cb*_562_ variant.^17*f*^ A cyt *cb*_562_ derivative with two additional Cys residues at its surface has been shown to assemble into a tetramer with a cryptand-like topology through selective formation of four interfacial disulfide bonds.^17*i*^ A computationally designed dimer of cyt *cb*_562_ has been arranged by Zn^2+^ coordination into 1D helical nanotubes, and 2D or 3D crystalline arrays.^6*b*^

Recently, we have constructed a heterodimeric Mb with two different active sites by modifying the interface of two protomers in the domain-swapped wild-type dimer,^18^ demonstrating that domain swapping may be utilized to design artificial heme proteins. In this study, we obtained domain-swapped cyt *cb*_562_ oligomers, and found that the two helices (helices 1 and 2) in the N-terminal region of one protomer and the other two helices (helices 3 and 4) in the C-terminal region of the other protomer interact in the dimer. In the crystal, three cyt *cb*_562_ dimers formed a unique cage structure with a Zn–SO_4_ cluster inside the cavity, showing that domain-swapping may be utilized to construct unique nanostructures.

## Results

### Formation and stability of dimeric cyt *b*_562_ and dimeric cyt *cb*_562_

*E. coli* cyt *b*_562_ solution changed its colour from red to black by an addition of acetic acid up to 40% (v/v) (pH 1.9) to its neutral pH solution, although no precipitation was observed. Cyt *b*_562_ has been reported to denature at pH 2.3.^19^ Therefore, the colour change was ascribed to the heme dissociation from the cyt *b*_562_ protein moiety in the presence of 40% (v/v) acetic acid, causing change in the heme coordination. After lyophilization of the cyt *b*_562_ solution, the protein powder was dissolved in 50 mM potassium phosphate buffer, pH 7.0, and analysed by size exclusion chromatography. A small peak was observed at ∼11.1 mL in the elution curve, in addition to the monomer peak at ∼12.7 mL (Fig. S1a†). We attributed the ∼11.1 mL peak to the dimer. The amount of the dimer was estimated at ∼10% from the area in the chromatogram. Approximately 45% and 86% of the dimers dissociated to monomers by incubation at 4 °C for 2 and 12 h, respectively (Fig. S2A†), showing that dimeric cyt *b*_562_ is relatively unstable.

Attachment of the heme to the protein moiety in cyt *b*_562_ may stabilize the dimer, since the holoMb dimer was more stable than the apoMb dimer.^11*c*^ To support this hypothesis, we used cyt *cb*_562_, in which the heme was covalently attached to the protein moiety through two thioether bonds by mutation of Arg98 and Tyr101 to Cys.^15,16b,c^ A large amount of oligomers was obtained by an addition of acetic acid to the cyt *cb*_562_ solution up to 40% (v/v) and subsequent dissolution of the obtained precipitate with buffer (Fig. S1b†). In contrast to dimeric cyt *b*_562_, dimeric cyt *cb*_562_ did not dissociate to monomers by incubation at 4 °C for 12 h (Fig. S2B†), showing that dimeric cyt *cb*_562_ is more stable than dimeric cyt *b*_562_.

### Optical absorption and circular dichroism measurements of dimeric cyt *cb*_562_

**Fig. 1:**
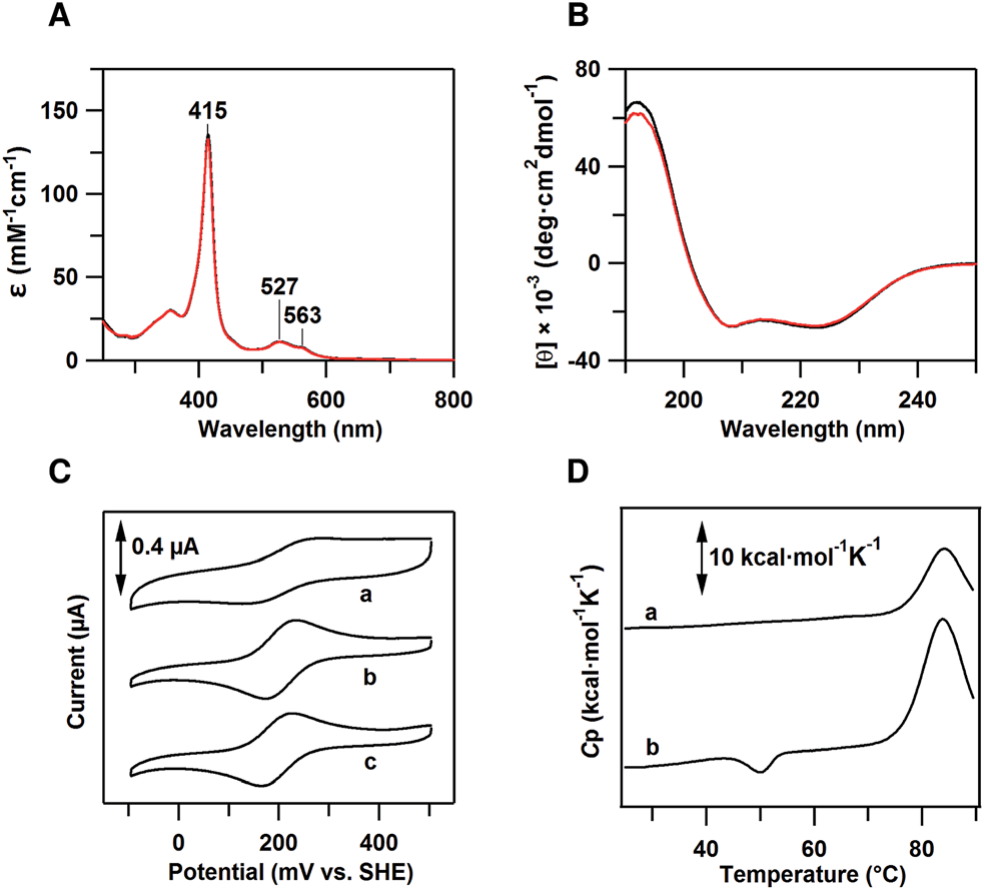
Characterization of dimeric cyt *cb*_562_. (A) Optical absorption spectra of oxidized monomeric (black) and dimeric (red) cyt *cb*_562_. (B) CD spectra of oxidized monomeric (black) and dimeric (red) cyt *cb*_562_. (C) Cyclic voltammograms of (a) monomeric *E. coli* cyt *b*_562_, (b) monomeric cyt *cb*_562_ and (c) dimeric cyt *cb*_562_. Scan rate was 50 mV s^–1^. (D) Differential scanning calorimetry thermograms of oxidized (a) monomeric and (b) dimeric cyt *cb*_562_. Scan rate was 1 °C min^–1^. Measurement conditions: sample concentration, (A) 10 μM, (B) 8 μM, (C) 200 μM and (D) 100 μM (heme unit); solvent, 50 mM potassium phosphate buffer; pH, 7.0.

The peak wavelength and intensity of the Soret and Q bands of oxidized dimeric cyt *cb*_562_ were similar to those of the oxidized monomer, showing that the active site structure was similar between the monomer and dimer (Fig. 1A). Similar negative Cotton effects at 208 and 222 nm, characteristic of an α-helical structure, were observed in the circular dichroism (CD) spectra of oxidized monomeric and dimeric cyt *cb*_562_, indicating that the secondary structures also do not change significantly by the dimerization (Fig. 1B). It has been shown in other heme proteins, *c*-type cyts and Mb, that the secondary structures are similar between the monomer and domain-swapped dimer.^11b–e^

### Dimeric cyt *cb*_562_ redox potential

Cyclic voltammetry (CV) was performed to determine the midpoint redox potential (*E*_m_) of dimeric cyt *cb*_562_ (Fig. 1C). The *E*_m_ value of the cyt *cb*_562_ monomer was obtained as 203 mV, similar to that reported previously (199–204 mV),^20^ and to that of cyt *b*_562_ (205 mV) (Fig. 1C). The *E*_m_ value of the cyt *cb*_562_ dimer (198 mV) was similar to that of the cyt *cb*_562_ monomer. Dimeric cyt *cb*_562_ exhibited a similar high redox potential as the monomer, suggesting that the active site structure of the dimer is similar to that of the monomer.

### Differential scanning calorimetry measurements of dimeric cyt *cb*_562_

Differential scanning calorimetry (DSC) measurements of oxidized monomeric and dimeric cyt *cb*_562_ were performed to investigate the thermodynamic properties of the dimer. A positive peak was observed at 83.9 °C in the DSC thermogram of the cyt *cb*_562_ monomer (Fig. 1D, a). We assigned this peak to denaturation of the cyt *cb*_562_ monomer, where the denaturation temperature of the cyt *cb*_562_ monomer was higher than that of the cyt *b*_562_ monomer (67.0 °C at pH 7.4; 73.8 °C at pH 5–6).^13*d*^ However, an additional small negative peak was observed at 50.1 °C in the DSC thermogram of the cyt *cb*_562_ dimer (Fig. 1D, b). No peak was observed in the thermogram around 50 °C after dissociation of the cyt *cb*_562_ dimer to monomers by heating the dimer solution up to 70 °C (Fig. S3†). Therefore, we attributed the small negative peak to the dissociation of the cyt *cb*_562_ dimer to monomers. The area of this peak in the thermogram corresponds to the enthalpy change (Δ*H*) during the dissociation. The Δ*H* value for the dissociation of the cyt *cb*_562_ dimer to monomers was obtained as –13 ± 2 kcal mol^–1^ (per dimer), showing that the dimer is enthalpically disfavoured compared to the monomer.

### Dimeric cyt *cb*_562_ crystal structure

**Fig. 2:**
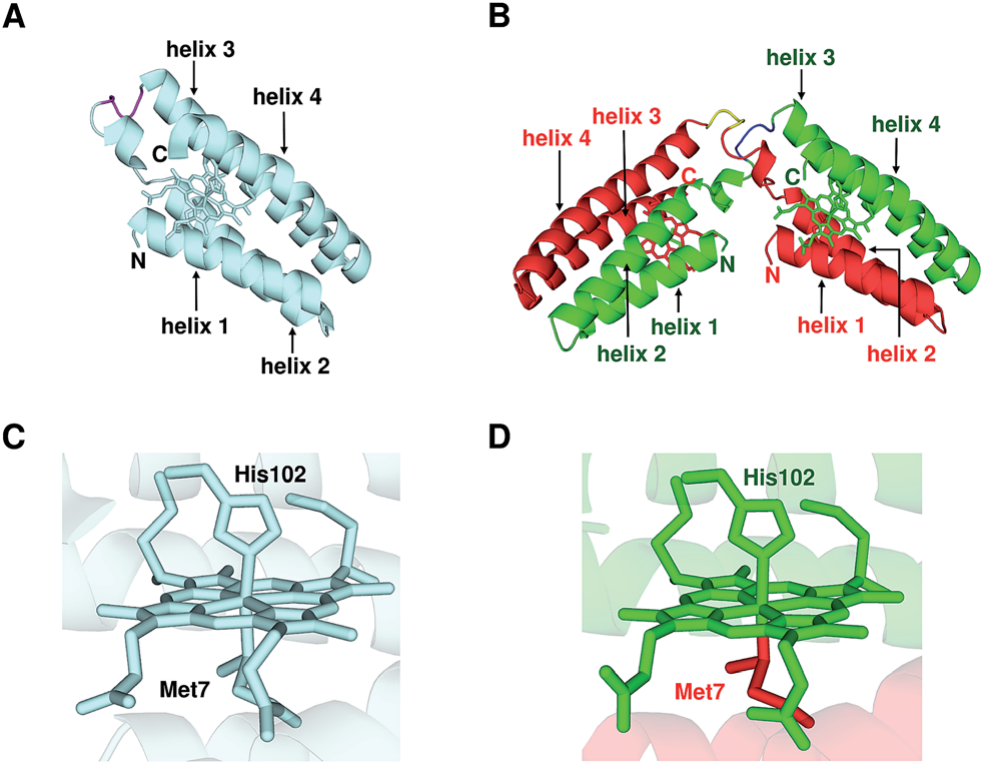
Crystal structures of oxidized monomeric and dimeric cyt *cb*_562_. (A) Protein structure of the monomer (K59W, R98C, and Y101C mutant of cyt *b*_562_; PDB ID: ; 2BC5). (B) Protein structure of the dimer solved in this study (PDB ID: ; 5AWI). (C) Active site structure of the monomer (PDB ID: ; 2BC5). (D) Active site structure of the dimer (PDB ID: ; 5AWI). The green and red regions in the dimer belong to different protomers. The hemes and the side chain atoms of heme-binding Cys residues (Cys98 and Cys101) and heme-coordinating residues (Met7 and His102) are shown as stick models. The N- and C-termini of the monomer and the protomers of the dimer are labelled as N and C, respectively. The α-helices are labelled helix 1, helix 2, helix 3 and helix 4 from the N-terminus. The hinge loop (Lys51–Asp54) is depicted in purple in the monomer, and blue and yellow in the dimer.

We performed X-ray crystallographic analysis to elucidate the detailed structure of dimeric cyt *cb*_562_. The 1.85 Å resolution structure of dimeric cyt *cb*_562_ exhibited a domain-swapped structure (PDB ID: ; 5AWI) (Fig. 2B). The two helices in the N-terminal region (helices 1 and 2) of a protomer and the other two helices in the C-terminal region (helices 3 and 4) of the other protomer interacted in the dimer. There were two independent cyt *cb*_562_ molecules forming a dimer in the asymmetric unit of the dimeric cyt *cb*_562_ crystal. The position of the backbone of the dimer overlapped well with that of the monomer (Fig. S4A†). We calculated the root-mean-square deviation (rmsd) for the Cα atoms between the structures of the monomer (Fig. 2A; K59W, R98C and Y101C mutant of cyt *b*_562_, PDB ID: ; 2BC5) and dimer. Residues in the N-terminal region (Ala1–Asp50) before a protomer hinge loop (Lys51–Asp54) and residues in the C-terminal region (Ser55–Arg106) after the hinge loop in the other protomer in the dimer were compared with the corresponding structural region of the monomer. The rmsd values were less than 0.36 Å, indicating that the corresponding structures of the monomer and dimer were similar. The heme orientation and the amino acid side chain positions at the active site of the dimer also overlapped well with those of the monomer (Fig. S4B†). Met7 and His102 were axially coordinated to the heme iron in the dimer as in the monomer (Fig. 2C), although Met7 originated from the other protomer to which the heme belonged (Fig. 2D).

**Fig. 3:**
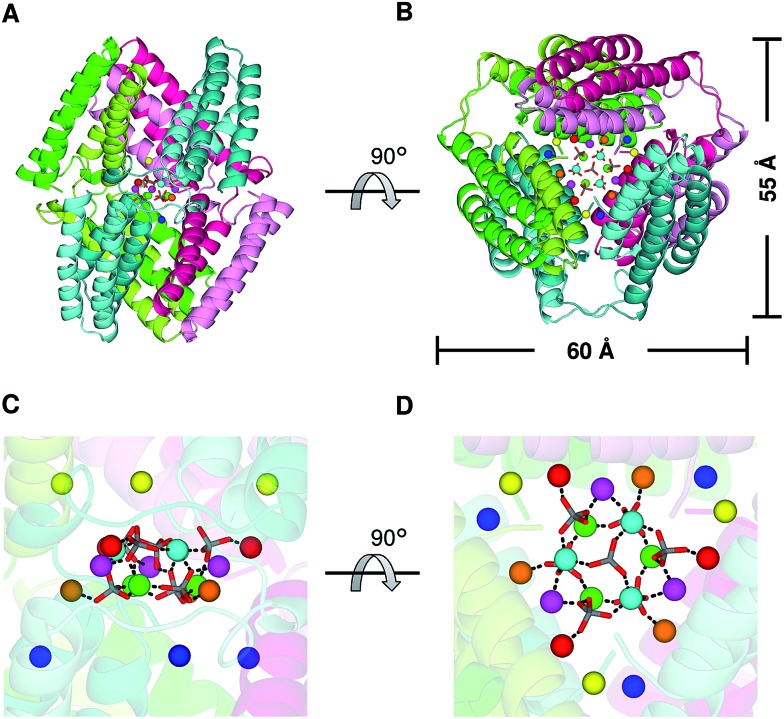
Cage structure constructed of three domain-swapped cyt *cb*_562_ dimers in the crystal (PDB ID: ; 5AWI): (A and B) overall structure of the cage. (B) is a 90°-rotated view of (A). The three dimers forming the cage are shown in combinations of green and light-green, blue-green and cyan, and red and pink, respectively. The horizontal scale bar (60 Å) corresponds to the distance between the α carbons (Cα) of the Pro56 residues in two protomers shown in green and pink. The vertical scale bar (55 Å) corresponds to the distance between the Cα of Pro53 in a protomer of one dimer (blue–green) and that of Thr96 in a protomer of another dimer (red). (C and D) Enlarged views of the Zn^2+^ and SO_4_^2–^ ions in the internal cavity. (D) is a 90°-rotated view of (C). The coordination bonds between Zn^2+^ and SO_4_^2–^ ions are shown as black dashed lines. The Zn^2+^ ions are shown as green, cyan, magenta, orange, red, yellow and blue spheres. The SO_4_^2–^ ions are shown as stick models, and their sulfur and oxygen atoms are coloured grey and red, respectively.

Three domain-swapped dimers of cyt *cb*_562_ formed a hollow cage-like structure in the crystal (Fig. 3A and B). The cage structure exhibited pseudo-*D*_3_ symmetry with one 3-fold and three pseudo 2-fold axes. The outer diameter of the cage was 55–60 Å (Fig. 3B). Interestingly, a cluster consisting of fifteen Zn^2+^ and seven sulfate (SO_4_^2–^) ions was encapsulated in the cage (Fig. 3C, D and S5†). The Zn^2+^ ions were bridged by the SO_4_^2–^ ions, and the side chains of Asp2, Glu4, Asp5 and Glu8 coordinated to the Zn^2+^ ions (Fig. S6A–S6E†). Although it is rare to observe SO_4_^2–^ ion-bridged Zn^2+^ ions in inorganic complexes,^21^ the Zn^2+^–SO_4_^2–^ coordination was apparently stabilized in the cyt *cb*_562_ cage by fixing the ion positions (Fig. S6†). The Zn^2+^ ions of the cluster are classified into five coordination patterns (Zn1–Zn5, three Zn ions for each pattern) (Fig. S6A–S6E†). Six additional Zn^2+^ ions (three Zn6 and three Zn7) were also observed inside the cavity (Fig. S5, S6F and S6G†). However, there was no hydrogen bond or hydrophobic interaction at the interface of the dimers in the crystal structure; thus, evidently the coordination of the amino acid side chains to the Zn^2+^ ions stabilized the cage structure.

### Interaction of dimeric cyt *cb*_562_ with Zn^2+^ ions in solution

**Fig. 4:**
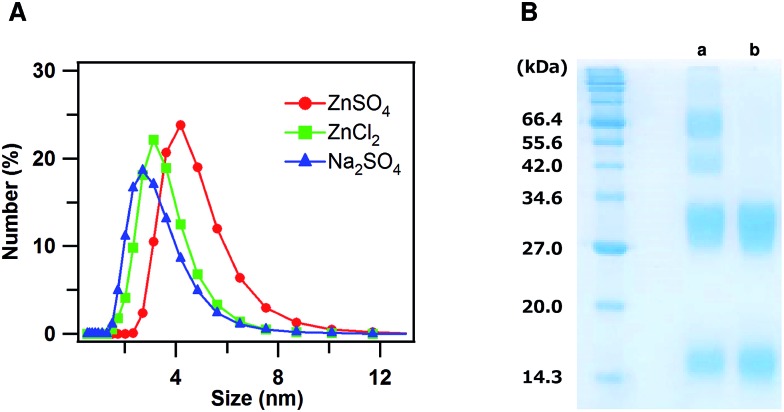
(A) Size distribution curves of oxidized dimeric cyt *cb*_562_ obtained from DLS measurements with an addition of ZnSO_4_ (circles, red), ZnCl_2_ (squares, green) or Na_2_SO_4_ (triangles, blue). Measurement conditions: sample concentration, 50 μM (heme unit); ZnSO_4_, ZnCl_2_, and Na_2_SO_4_ concentration, 300 μM; solvent, 15 mM MES buffer; pH 5.5; temperature, 25 °C. (B) SDS-PAGE analysis after the cross-linking reaction of the oxidized dimeric cyt *cb*_562_ solution with an addition of ZnSO_4_ (a) or Na_2_SO_4_ (b). Dimeric cyt *cb*_562_ (heme unit, 200 μM) was reacted with BS3 (25 mM) after an addition of ZnSO_4_ or Na_2_SO_4_ (1.2 mM) at pH 7.5.

To investigate the interaction of dimeric cyt *cb*_562_ with Zn^2+^ ions in solution, dynamic light scattering (DLS) measurements of oxidized dimeric cyt *cb*_562_ were performed with an addition of ZnSO_4_, ZnCl_2_ and Na_2_SO_4_ at pH 5.5, at which the crystal was obtained (Fig. 4A). The mean diameter of the particles became larger for dimeric cyt *cb*_562_ with an addition of ZnSO_4_ (Zn^2+^ and SO_4_^2–^ ions) (4.6 ± 0.3 nm) compared to that with an addition of ZnCl_2_ (Zn^2+^ and Cl^–^ ions) (3.4 ± 0.4 nm) or Na_2_SO_4_ (Na^+^ and SO_4_^2–^ ions) (3.1 ± 0.6 nm), indicating that the dimers interacted among each other in solution in the presence of Zn^2+^ and SO_4_^2–^ ions, although a defined cage structure was not constructed.

We added bis-sulfosuccinimidyl suberate (BS3) to the oxidized dimeric cyt *cb*_562_ solution and cross-linked cyt *cb*_562_ to investigate the intermolecular interaction of cyt *cb*_562_ in solution in more detail. After the reaction of the cyt *cb*_562_ dimers with BS3 in the presence of Zn^2+^ and SO_4_^2–^ ions, the solution was analysed by SDS-PAGE (Fig. 4B, a). Four bands were detected in the SDS-PAGE gel at about 15, 30, 45 and 65 kDa, which corresponded well to the molecular weights of the monomer, dimer, trimer and tetramer, respectively. The dimers may have dissociated to monomers during the cross-linking reaction, resulting in formation of trimers. A broad band was also detected in the SDS-PAGE gel at a molecular weight higher than 100 kDa. When cross-linking the dimer in the presence of Na^+^ and SO_4_^2–^ ions, only two bands were detected in the SDS-PAGE gel at about 15 and 30 kDa, corresponding to the cyt *cb*_562_ monomer and dimer, respectively (Fig. 4B, b). These results showed that cyt *cb*_562_ forms oligomers larger than a tetramer in solution in the presence of Zn^2+^ and SO_4_^2–^ ions, but not in the absence of them.

## Discussion

Domain swapping has been observed in various heme proteins.^9a,b,f,h,11^ In *c*-type cyts and Mb, the active sites of the domain-swapped oligomers are constructed with two protomers.^11^ The heme is coordinated with the amino acids from different protomers in dimeric *c*-type cyts,^11a,b,d,e^ which may stabilize the domain-swapped structure. In cyt *cb*_562_, less domain-swapped dimers dissociated to monomers at 4 °C compared to cyt *b*_562_ (Fig. S2†). It has been reported that the folding free energy change of oxidized cyt *cb*_562_ (K59W, R98C and Y101C cyt *b*_562_ mutant) is –42 kJ mol^–1^, whereas that of oxidized cyt *b*_562_ is –30 kJ mol^–1^.^16*b*^ The folding free energy change of oxidized HT cyt *c*_552_ exhibits a greater negative value than that of oxidized horse cyt *c* (HT cyt *c*_552_, –75 kJ mol^–1^; horse cyt *c*, –23 kJ mol^–1^ (at 25 °C, pH 7)),^22^ whereas the dissociation temperature of the domain-swapped HT cyt *c*_552_ dimer (92 °C)^11*b*^ is higher than that of the horse cyt *c* dimer (58 °C).^11*a*^ Since a domain-swapped dimer shares a similar three-dimensional structure with its monomer, excluding the hinge loop, the folding free energy change of the dimer may correlate with that of the monomer. In fact, dissociation of a domain-swapped dimer to monomers has been suggested to occur *via* significant unfolding of the polypeptide for many proteins, including RNase A, cyanovirin-N, Stefin A, M^Pro^-C and p13suc1.^23^ Taking these results into consideration, stabilization of the domain-swapped dimer on dissociation to monomers may correspond to the negative value in the folding free energy change of its monomeric protein.

The Δ*H* value for the dissociation of the cyt *cb*_562_ dimer to monomers was obtained as –13 kcal mol^–1^ (per dimer) (Fig. 1D). The Δ*H* values for dimer dissociation varies from negative to positive values in *c*-type cyt proteins. The Δ*H* value of the horse cyt *c* dimer has been reported to be –40 kcal mol^–1^ (per dimer),^11*a*^ whereas that of the PA cyt *c*_551_ dimer, HT cyt *c*_552_ dimer and *Aquifex aeolicus* cyt *c*_555_ dimer are ∼0, +14 and –14 kcal mol^–1^ (per dimer), respectively.^11b,d,e^ Although many other factors including solvation may affect the Δ*H* value, the number of hydrogen bonds at the hinge loop decreased in cyt *cb*_562_ by the dimerization (dimer, three and six hydrogen bonds for each protomer; monomer, seven hydrogen bonds) (Fig. S7†).^24^ This decrease in the hydrogen bond number in cyt *cb*_562_ by the dimerization may contribute to the negative Δ*H* value.

**Fig. 5:**
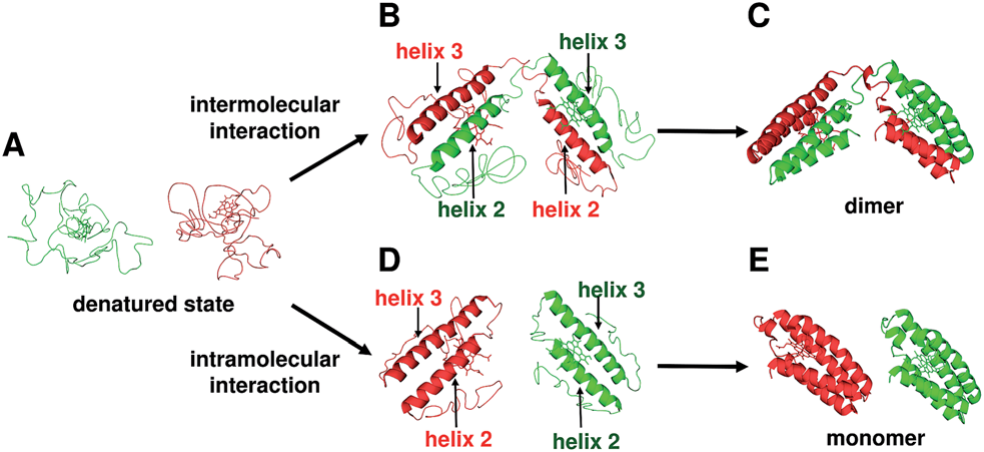
Schematic view of the dimerization process of cyt *cb*_562_ during folding. (A) Unfolded cyt *cb*_562_. (B) Dimer intermediate with intermolecular interaction between helices 2 and 3. (C) Dimeric cyt *cb*_562_ (PDB ID: ; 5AWI). (D) Monomer intermediates with intramolecular interaction between helices 2 and 3. (E) Monomeric cyt *cb*_562_ (PDB ID: ; 2BC5).

The two helices in the N-terminal region (helices 1 and 2) of a protomer and the other two helices in the C-terminal region (helices 3 and 4) of the other protomer interacted between each other in domain-swapped dimeric cyt *cb*_562_ (Fig. 2B). Although the heme active site was constructed with two protomers and the axial ligands of the heme originated from different protomers in the dimer, the active site structures were similar between the monomer and dimer (Fig. 2C, D and S4B†). In agreement with the structural results, the absorption spectrum and redox potential were similar between the cyt *cb*_562_ monomer and its domain-swapped dimer (Fig. 1A and C). We have previously shown that domain-swapped oligomers of horse cyt *c* form through intermolecular hydrophobic interaction between the N- and C-terminal α-helices at the early stage of folding.^25^ Recently, it has been shown by folding simulation that apoMb adopts a similar domain swapping mechanism to that of horse cyt *c*, and the intermolecular contacts between the helices A–B region of one molecule and the helices G–H region of the another molecule at the early stage of folding result in formation of a domain-swapped dimer.^26^ In addition, it has been suggested that helices 2 and 3 form first at the initial stage of folding in wild-type apo cyt *b*_562_ and its mutant, in which the hydrophobic residues were replaced with Asp and Gly.^13c,27^ Cyt *cb*_562_ were precipitated by the addition of acetic acid (40% (v/v)), and presumably refolded by dissolution of the precipitate with buffer (Fig. S1b†). The present result, where the loop between helices 2 and 3 served as the hinge loop for domain swapping in cyt *cb*_562_, is consistent with the previously mentioned feature of helices 2 and 3 forming at the initial folding stage of cyt *cb*_562_ monomer. The domain-swapped dimer forms when helices 2 and 3 interact intermolecularly at the initial stage of folding; whereas, the intramolecular interaction of helices 2 and 3 results in formation of monomers (Fig. 5). However, the intermediate complex of cyt *cb*_562_ with intermolecular interaction between helices 2 and 3 is likely short-lived, since its folding has been reported to proceed without formation of stable intermediates.^16*c*^

A cage structure of cyt *cb*_562_ was formed by three dimers in the crystal (Fig. 3A), although defined cages were not obtained in solution (Fig. 4B). However, the dimeric cyt *cb*_562_ crystal was not obtained without Zn^2+^ ions. The carboxylate groups of Asp (Asp2, Asp5, Asp12 and Asp39) and Glu (Glu4 and Glu8), together with the amino and carbonyl groups of Ala1, were coordinated to Zn^2+^ ions in the cavity of the domain-swapped cyt *cb*_562_ cage (Fig. S6†). The coordination of the amino acids to the Zn^2+^ ions guided the three cyt *cb*_562_ dimers into a cage structure, whereas no hydrogen bond or hydrophobic interaction was detected at the dimer interfaces. It has been reported that crystallization of a protein is promoted by increase in symmetry with the introduction of a Zn^2+^ coordination site, owing to the strict symmetry imposed on the protein molecule.^28^ The Zn^2+^ ions may have enhanced crystallization of the cyt *cb*_562_ dimer, where the cage structure of dimeric cyt *cb*_562_ exhibited a pseudo-*D*_3_ symmetry.

Although protein cages are potentially useful for many applications, such as drug and gene carriers,^29^ molecular flasks,^30^ nanomedicines,^31^ and nanodevices,^32^ successful protein cage assemblies are limited.^4,8f,g^ Ni and Tezcan reported a cyt *cb*_562_ surface mutant crystalline cage, with cage cavity size around 35 Å in diameter, and success in encapsulating a heme peptide fragment inside the cage.^17*f*^ We obtained a smaller cage constructed with three domain-swapped cyt *cb*_562_ dimers encapsulating a Zn–SO_4_ cluster (fifteen Zn^2+^ and seven SO_4_^2–^ ions). The cavity size of the domain-swapped cyt *cb*_562_ cage was calculated as 1860 Å^3^ by the program, VOIDOO,^33^ using a probe radius of 1.4 Å and excluding the Zn^2+^ and SO_4_^2–^ ions in the model; whereas, it was 32 740 Å^3^ for the cage structure of the cyt *cb*_562_ surface mutant. The interaction of the Zn^2+^ ions through the SO_4_^2–^ ions may be a factor in the small cavity size of the domain-swapped cyt *cb*_562_ cage.

Three Zn^2+^ ion binding sites have been detected in the cyt *cb*_562_ surface mutant cage.^17*f*^ The Zn^2+^ ion coordinating non-modified residues (Ala1, Glu8, Asp12 and Asp39) in the surface mutant cage^17*f*^ also coordinated to the Zn^2+^ ions in the domain-swapped dimer cage. Especially, the Zn7 site with Ala1, Asp12 and Asp39 of the domain-swapped dimer cage corresponded well to the Zn^2+^ ion binding site with Ala1 and Asp39 of the surface mutant cage (Fig. S8†). This indicates that N-terminal Ala1 and Asp39 have a tendency to interact together with Zn^2+^ ions.

Monomeric cyt *cb*_562_ did not crystalize under similar conditions that yielded a crystal for the dimer, although the secondary structures of the monomer and dimer were similar (Fig. S4A†). Therefore, the domain-swapped structure may have an important role in construction of the cage structure for dimeric cyt *cb*_562_. The amino acid residues in the dimer need to coordinate to the Zn^2+^ ions at appropriate positions to form the cage structure. Since the two four-helix bundle units were connected by a loop (hinge loop) in dimeric cyt *cb*_562_, the relative positions of the two units could be adjusted, whereas the entropy loss on cage formation by three domain-swapped dimers may be smaller than that by six monomers. These properties may have guided the dimers to form a cage structure.

## Conclusions

Oligomers were obtained for cyt *b*_562_ and cyt *cb*_562_ by treatment with acetic acid. Dimeric cyt *cb*_562_ was more stable than dimeric cyt *b*_562_ at 4 °C, showing that the dimer stability increases by the heme attachment to the protein moiety. According to X-ray analysis, dimeric cyt *cb*_562_ exhibited a domain-swapped structure, where the two helices in the N-terminal region (helices 1 and 2) in a protomer and the other two helices in the C-terminal region (helices 3 and 4) of the other protomer interacted between each other. These results were in agreement with the previously mentioned proposal that helices 2 and 3 form at the initial cyt *cb*_562_ folding stage. Three dimeric cyt *cb*_562_ formed a cage structure containing a Zn–SO_4_ cluster inside the cavity in the crystal. Although no hydrogen bond or hydrophobic interaction existed at the interfaces of the dimers, coordination of the amino acids of cyt *cb*_562_ to Zn^2+^ ions directed formation of the cage structure. The cavity size of the domain-swapped cyt *cb*_562_ cage was smaller than that of the cyt *cb*_562_ surface mutant cage, where the interaction of the Zn^2+^ ions through the SO_4_^2–^ ions was important for maintaining the small domain-swapped cyt *cb*_562_ cage. The connection of two four-helix bundle units through the hinge loop in the domain-swapped dimer apparently stabilized the cage structure. These results show that domain swapping may be useful for designing artificial proteins with unique nanostructures, by connecting structural units with a conformation-adjustable loop.
