# Supplementary material for: Domain-swapped cytochrome cb 562 dimer and its nanocage encapsulating a Zn–SO4 cluster in the internal cavity
Source: Chem Sci. 2015 Sep 22;6(12):7336–42. doi: 10.1039/c5sc02428e (PMC5519777; doi:10.1039/c5sc02428e)
Supplement: Supplementary file 1 [file SC-006-C5SC02428E-s001.pdf]

Electronic Supplementary Material (ESI) for Chemical Science.
This journal is © The Royal Society of Chemistry 2015

Electronic Supplementary Information

Domain-swapped Cytochrome *cb*$_{562}$ Dimer and Its Nanocage Encapsulating a Zn-SO_4 Cluster in the Internal Cavity

Takaaki Miyamoto,[a] Mai Kuribayashi,[a] Satoshi Nagao,[a] Yasuhito Shomura,[b] Yoshiki Higuchi,[cd] and Shun Hirota*[a]

[a] *Graduate School of Materials Science, Nara Institute of Science and Technology, 8916-5 Takayama, Ikoma, Nara 630-0192, Japan*

[b] *Graduate School of Science and Engineering, Ibaraki University, 4-12-1, Nakanarusawa, Hitachi, Ibaraki 316-8511, Japan*

[c] *Department of Life Science, Graduate School of Life Science, University of Hyogo, 3-2-1 Koto, Kamigori-cho, Ako-gun, Hyogo 678-1297, Japan*

[d] *RIKEN SPring-8 Center, 1-1-1 Koto, Sayo-cho, Sayo-gun, Hyogo 679-5148, Japan*

Contents

1. Experimental section p. S2–S5

2. Supplementary figures and table

Fig. S1 Size exclusion chromatograms of oxidized *E. coli* cyt *b*$_{562}$ and cyt *cb*$_{562}$. p. S6

Fig. S2 Size exclusion chromatograms of the solution containing dimeric cyt *b*$_{562}$ and dimeric cyt *cb*$_{562}$. p. S7

Fig. S3 Differential scanning calorimetry thermograms of dimeric cyt *cb*$_{562}$. p. S8

Fig. S4 Overlapped view of the protein and active site structures of monomeric and dimeric cyt *cb*$_{562}$. p. S9

Fig. S5 $F_o - F_c$ omit map of the Zn^{2+} ions in the internal cavity of the cage structure of dimeric cyt *cb*$_{562}$. p. S10

Fig. S6 Coordination structures of Zn^{2+} and SO_4^{2-} ions in the internal cavity of the cage structure of dimeric cyt *cb*$_{562}$. p. S11

Fig. S7 Schematic views of the hydrogen bonds at the hinge loop for the protomers of dimeric cyt *cb*$_{562}$ and monomeric cyt *b*$_{562}$. p. S12

Fig. S8 Comparison of Zn binding sites containing Ala1 and Asp39 between the domain-swapped cyt *cb*$_{562}$ dimer cage and cyt *cb*$_{562}$ surface mutant cage. p. S13

Table S1 Crystallographic statistics of data collection and structure refinement of dimeric cyt *cb*$_{562}$. p. S14

3. Supplementary references p. S15

1. Experimental section

Plasmids of *E. coli* cyt b_{562} and cyt cb_{562}

The *E. coli* cyt b_{562} gene was synthesized (Life Technologies Japan, Tokyo) and sub-cloned into the *Nde*I-*BamH*I site of the pET29b plasmid. Conversion of cyt b_{562} to cyt cb_{562} (R98C/Y101C cyt b_{562} mutant) was based on reported method.[1] The cyt cb_{562} gene was sub-cloned into the *EcoR*I-*Hind*III site of the pKK223-3 plasmid. DNA sequencing was carried out with a BigDye Terminator v3.1 cycle sequencing kit (Applied Biosystems, Inc., Foster City, CA) with an ABI PRISM 310 genetic analyzer sequencing system (Applied Biosystems, Inc.). The obtained plasmids of cyt b_{562} and cyt cb_{562} were introduced into competent cells of *E. coli* BL21 (DE3) (Novagen) and *E. coli* JCB387, respectively. Cyt cb_{562} was co-expressed with the cyt *c* maturation (ccm) proteins by introducing the pEC86 plasmid into the *E. coli* JCB387 competent cells.[2]

Purification of *E. coli* cyt b_{562} and cyt cb_{562}

E. coli BL21 (DE3) cells overproducing *E. coli* cyt b_{562} were grown in LB broth at 37 °C. Isopropyl β-D-1-thiogalactopyranoside was added to the *E. coli* solution (final concentration, 0.25 mM) when the OD_{600} value reached 0.4–0.5. The OD_{600} value reached about 1.6 when culturing the *E. coli* solution at 37 °C for 3 h. The cells were harvested by centrifugation, and subsequently suspended in a minimal volume of 50 mM potassium phosphate buffer, pH 7.0. Cyt b_{562} was extracted from the cells by freeze-thawing. After estimating the amount of cyt b_{562} from the absorbance at 280 nm, more than 1 equivalent (against cyt b_{562}) of hemin in 1 M NaOH was added to the solution. To remove the extra hemin, the protein solution was centrifuged and the obtained supernatant was passed through an anion exchange column with DE52 (Whatman). The protein solution was dialyzed overnight in 10 mM potassium phosphate buffer, pH 4.8. Cyt b_{562} was purified by cation exchange chromatography with CM52 (Whatman) and gel filtration chromatography (Hiload 26/60 Superdex 75, GE Healthcare, Buckinghamshire) using a FPLC system (BioLogic DuoFlow 10, Bio-Rad, CA) (flow rate, 0.8 mL/min; monitoring wavelength, 418 nm; solvent, 50 mM potassium phosphate buffer, pH 7.0; temperature, 4 °C). Cyt b_{562} was oxidized by an addition of ten equivalents of potassium ferricyanide, and the ferricyanide ion was removed from the cyt b_{562} solution with the DE52 column (Whatman). The purity of cyt b_{562} was confirmed by the ratio of the absorbance at 418 nm to that at 280 nm ($Abs_{418}/Abs_{280} > 6$). The concentration of cyt b_{562} was adjusted by the intensity of its Soret band using the molar extinction coefficient for cyt b_{562} reported previously (ε_{418} = 117.4 $mM^{-1}cm^{-1}$).[3]

For cyt cb_{562}, *E. coli* JCB387 cells overexpressing cyt cb_{562} were cultured in LB broth at 37 °C for 16 h until the OD_{600} value reached about 2.0. The cells were harvested by centrifugation, and subsequently suspended in a minimum volume of 50 mM potassium

phosphate buffer, pH 7.0. The pellets were suspended in spheroplasting buffer (10 mM Tris-HCl buffer, pH 8.0, containing 10 mM EDTA and 20% (w/v) sucrose) at 4 °C. After the obtained solution was incubated at 4 °C for 10 min, the precipitate was collected by centrifugation. Cold pure water was added to the pellet containing cyt cb_{562}. The obtained cyt cb_{562} solution was mixed on ice, and subsequently centrifuged. After the buffer of the supernatant was exchanged by dialysis to 25 mM sodium acetate buffer, pH 5.0, cyt cb_{562} was purified by cation exchange chromatography with CM52 (Whatman). The buffer of the cyt cb_{562} solution was exhanged by dialysis to 10 mM Tris-HCl buffer, pH 8.0, and cyt cb_{562} was purified by anion exchange chromatography (UNO-Q, Bio-Rad) and subsequently by gel filtration chromatography (Hiload 26/60 Superdex 75, GE Healthcare) using the FPLC system (BioLogic DuoFlow 10, Bio-Rad) (flow rate, 1.0 mL/min; monitoring wavelength, 415 nm; solvent, 10 mM Tris-HCl buffer, pH 8.0 (for anion exchange chromatography) and 50 mM potassium phosphate buffer, pH 7.0 (for gel filtration chromatography); temperature, 4 °C). Cyt cb_{562} was oxidized by the same procedures as cyt b_{562}. The purity of cyt cb_{562} was confirmed by the ratio of the absorbance at 415 nm to that at 280 nm ($Abs_{415}/Abs_{280} > 8$). Molar extinction coefficients of oxidized monomeric and dimeric cyt cb_{562} were determined by the hemochrome method as $\varepsilon_{415} = 136 \pm 1$ and $\varepsilon_{415} = 133 \pm 1$ $mM^{-1}cm^{-1}$, respectively.[4] The concentration of cyt cb_{562} was adjusted by the intensity of its Soret band.

Preparation of dimeric cyt *b*₅₆₂ and dimeric cyt *cb*₅₆₂

Oxidized dimeric *E. coli* cyt b_{562} and dimeric cyt cb_{562} were prepared by an addition of 75% (v/v) acetic acid to oxidized *E. coli* cyt b_{562} and cyt cb_{562} (1 mM), respectively, up to 40% (v/v) in 50 mM sodium phosphate buffer, pH 7.0, at 37 °C. The protein solution was lyophilized to remove acetic acid. The obtained precipitate was dissolved in 50 mM sodium phosphate buffer, pH 7.0, and the obtained protein solution was filtered (pore size, 0.45 μm; Millex, Millipore, Bedford, USA). Dimeric cyt b_{562} and dimeric cyt cb_{562} were purified by gel filtration chromatography (HiLoad 26/60 Superdex75, GE healthcare, Buckinghamshire) using the FPLC system (BioLogic DuoFlow 10, Bio-Rad) (flow rate, 1.0 mL/min; monitoring wavelength, 418 nm [cyt b_{562}) and 415 nm [cyt cb_{562}]; solvent, 50 mM potassium phosphate buffer, pH 7.0, 4 °C). The fractions containing dimeric cyt cb_{562} were incubated at 37 °C for 1 h to remove the unstable dimers. After the incubation, dimeric cyt cb_{562} was purified using the same column and FPLC system described above.

Size exclusion chromatography analysis

The amount of oxidized dimeric cyt b_{562} and cyt cb_{562} in each solution was analyzed by size exclusion chromatography with a Superdex 75 10/300 GL gel column (GE Healthcare) using the FPLC system (BioLogic DuoFlow 10, Bio-Rad) (flow rate, 0.5 mL/min; monitoring wavelength, 418 nm [cyt b_{562}] and 415 nm [cyt cb_{562}]; solvent, 50 mM potassium phosphate

buffer, pH 7.0; temperature, 4 °C). The elution curves were fitted with a multi-peak Gaussian fitting procedure (Origin 8, OriginLab Corporation). The percentages of the monomer and dimer were obtained by dividing the area of the peak by the total area of the peaks in the elution curve.

Optical absorption and CD measurements

Absorption spectra were measured with a UV-2450 spectrophotometer (Shimadzu, Kyoto, Japan) using a 1 cm path-length quartz cell at 20 °C. CD spectra were measured with a J-725 CD spectrophotometer (Jasco, Japan) using a 0.1 cm path-length quartz cell at 20 °C. Sample solutions of oxidized monomeric and dimeric cyt cb_{562} were prepared in 50 mM potassium phosphate buffer, pH 7.0. The heme concentration of the sample solution was adjusted to 10 and 8 μM for the absorption and CD measurement, respectively.

Electrochemistry

CV responses were obtained with an ALS-612DN electrochemical analyzer (BAS Inc., Tokyo, Japan). An Au electrode was used as a working electrode. A Pt wire and Ag/AgCl (3 M NaCl) were used as counter and reference electrode, respectively. Modification of the surface of the Au electrode was performed by the following procedure. The Au electrode was polished with 0.05 μm alumina water slurry, rinsed with pure water to remove residual organic compounds from the electrode surface, and cleaned by electrochemical oxidation/reduction treatment.[5] The Au electrode was dipped in pure water containing 20 mM 2-mercaptoethanol (Wako, Osaka, Japan) for 1 h, and subsequently rinsed with pure water.[6] Cyclic voltammograms of oxidized monomeric and dimeric cyt cb_{562} (heme unit, 200 μM) were recorded in 50 mM sodium phosphate buffer, pH 7.0, containing 100 mM $MgCl_2$. All measurements were performed with a scan rate of 50 mV/s at room temperature, after degassing with a vacuum line and flowing Ar gas for at least 5 min to remove oxygen from the solution. The potentials are reported with respect to the standard hydrogen electrode.

Differential scanning calorimetric measurements

DSC thermograms of oxidized monomeric and dimeric *E. coli* cyt cb_{5b2} (heme unit, 100 μM) were measured using VP-DSC (MicroCal, GE Healthcare) at a scan rate of 1 °C/min with 50 mM potassium phosphate buffer, pH 7.0.

X-ray crystallography

Crystallization of dimeric cyt cb_{562} was carried out at 4 °C using the sitting drop vapor diffusion method. Protein concentration was adjusted to 24 mg/mL in 50 mM Tris-HCl buffer, pH 7.0. The droplet prepared by mixing 1 μL of the protein solution with 1 μL of the reservoir solution was equilibrated. The reservoir solution consisted of 25% PEG MME 350, 6 mM $ZnSO_4$, and

750 mM MES buffer, pH 5.5. A crystal was observed in the protein solution after incubation at 4 °C for a week.

Diffraction data were collected at the BL38B1 beamline at SPring-8, Japan, using a Quantum315 detector (ADSC). The crystal was mounted on a cryo-loop without an additional cryoprotectant, and flash-frozen at 100 K in a nitrogen cryo system. The crystal-to-detector distance was 220 mm, and the wavelength was 1.0000 Å. The oscillation angle was 0.5°, and the exposure time was 4 s per frame. The total number of frames was 270. The diffraction data were processed using the program, HKL2000.[7] The preliminary structure was obtained by a molecular replacement method (MOLREP) using the atomic coordinates of the structure of an *E. coli* cyt cb_{562} monomer (K59W, R98C, and Y101C mutant of cyt b_{562}; PDB ID: 2BC5) as a starting model. The structure refinement was performed using the program, REFMAC.[8] The molecular model was manually corrected, and water molecules were picked up in the electron density map using the program, COOT. The data collection and refinement statistics are summarized in supplemental Table S1. The cavity size was calculated by the program, VOIDOO, using a probe radius of 1.4 Å.[9]

Dynamic light scattering

DLS experiments were performed with a Zetasizer Nano ZS analyzer (Malvern, Worcestershire, UK). Oxidized monomeric and dimerc cyt cb_{562} solutions (50 µM, heme unit) in 15 mM MES buffer, pH 5.5, containing 300 µM $ZnSO_4$, $ZnCl_2$, or Na_2SO_4, were incubated at room temperature for 1.5 h, and filtered (pore size, 0.45 µm; Millex, Millipore) before measurements. Each measurement was performed eight times at 25 °C and averaged.

Cross-linking of cyt cb_{562}

Oxidized dimerc cyt cb_{562} (heme unit, 200 µM) was reacted with BS3 (25 mM) in 15 mM MES buffer, pH 7.5, containing 1.2 mM $ZnSO_4$ or Na_2SO_4. After incubation of the reaction solution at 25 °C for 30 min, the reaction was quenched by an addition of 1 M Tris-HCl buffer, pH 8.0 (final Tris concentration, 50 mM). The reaction mixture was analyzed by SDS-PAGE and size exclusion chromatography using a Superdex 200 26/600 GL gel column (GE Healthcare) with the FPLC system (AKTAPrime Plus, GE Healthcare, UK) (flow rate, 1.0 mL/min; monitoring wavelength, 280 nm; solvent, 25 mM Tris-HCl buffer, pH 8.0, containing 150 mM NaCl; temperature, 4 °C). The fractions eluted from the column were collected and analysed by MALDI-TOF MS spectroscopy (Autoflex II, Bruker Daltonics, USA) using sinapinic acid as a matrix in linear mode. The buffer of the sample was exchanged to pure water using an Amicon ultrafiltration tube (Millipore, Bedford, USA) before mass measurements.

2. Supplementary figures and table

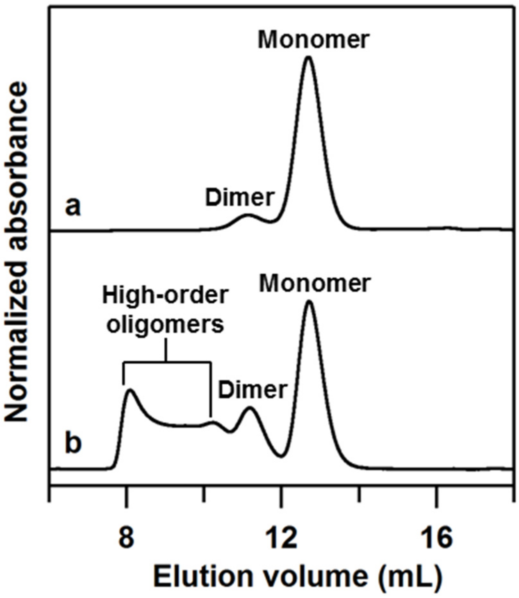

Fig. S1 Size exclusion chromatograms of oxidized (a) *E. coli* cyt b_{562} and (b) cyt cb_{562}. Acetic acid was added to the protein solution up to 40% (v/v), followed by lyophilization and dissolution with 50 mM phosphate buffer, pH 7.0. Monitoring wavelengths were 418 and 415 nm for cyt b_{562} and cyt cb_{562}, respectively. The intensities of the curves are normalized by the maximum absorbance.

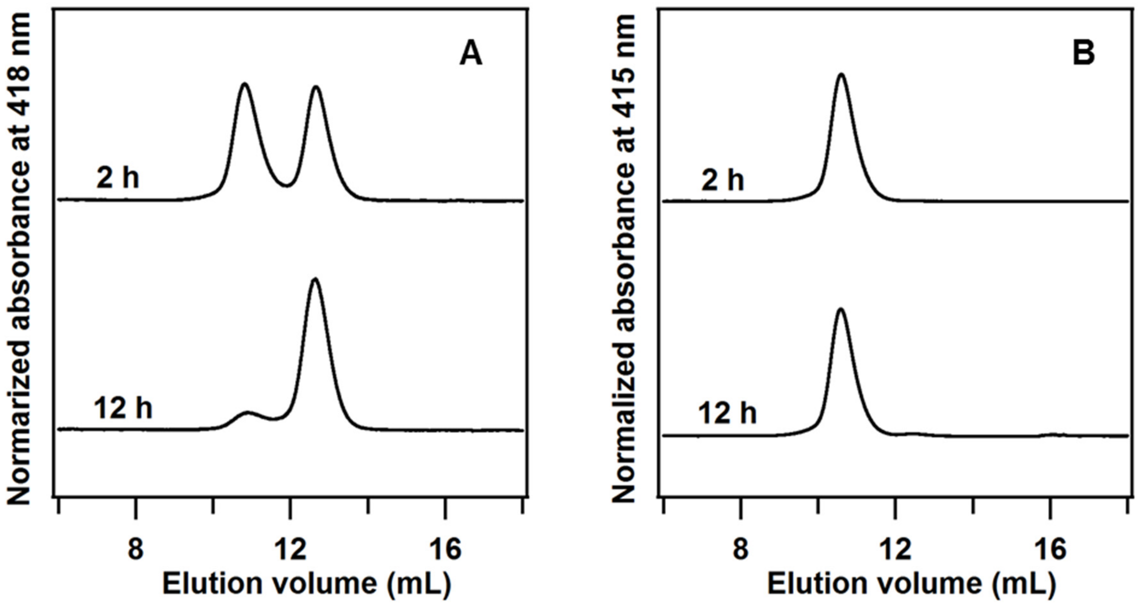

Fig. S2 Size exclusion chromatograms of the solution containing (A) dimeric cyt b_{562} and (B) dimeric cyt cb_{562}. The solution containing the dimer was analyzed after incubation at 4 °C for 2 h and 12 h. The intensities of the curves are normalized by the total area of the curve.

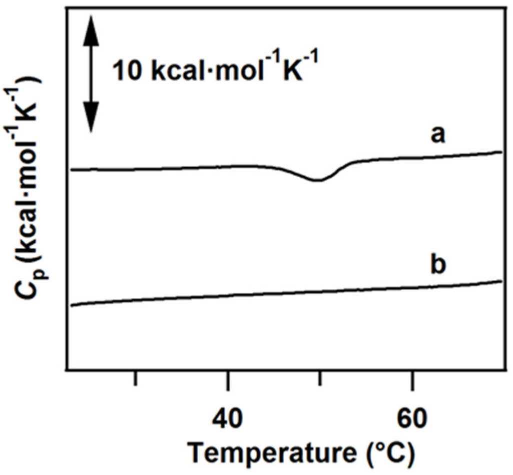

Fig. S3 Differential scanning calorimetry thermograms of dimeric cyt *cb*$_{562}$. The (a) first and (b) second scans from 20 to 70 °C. Measurement conditions: sample concentration, 100 μM (heme unit); solvent, 50 mM potassium phosphate buffer; pH, 7.0.

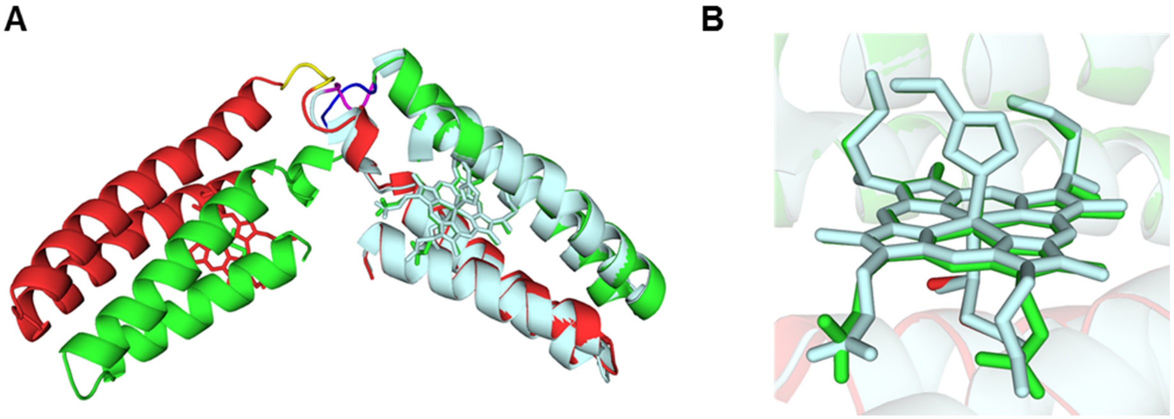

Fig. S4 Overlapped views of the (A) protein and (B) active site structures of monomeric (PDB ID: 2BC5) (pale-cyan) and dimeric (PDB ID: 5AWI) (green and red) cyt *cb*$_{562}$. Met7, Cys98, Cys101, His102, and the hemes are shown as stick models.

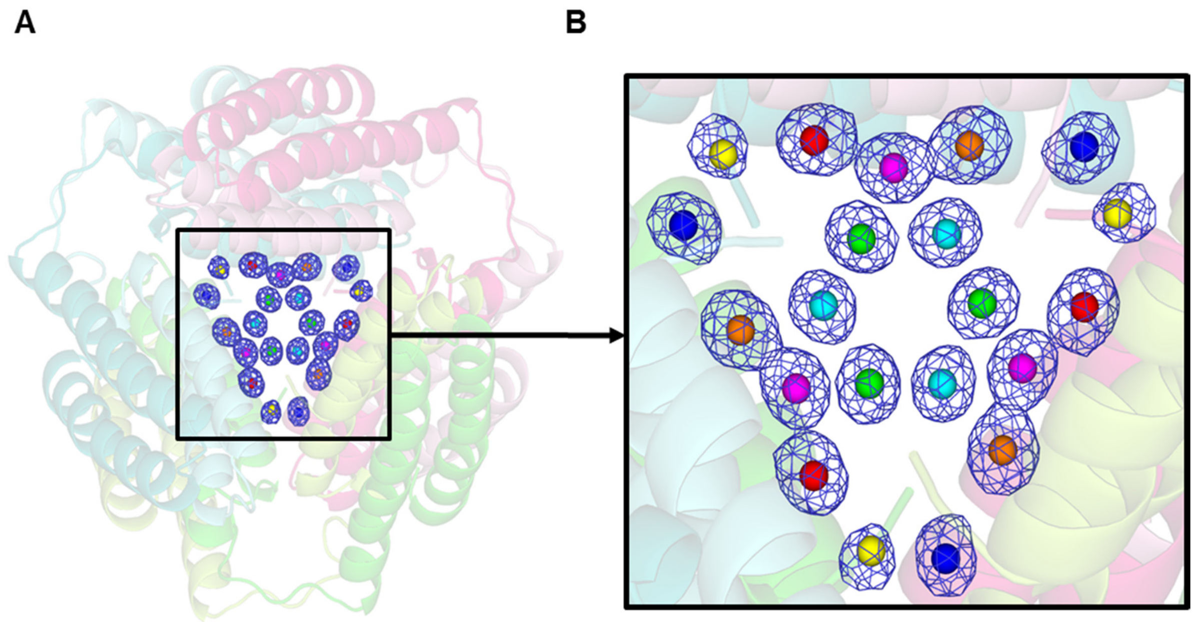

Fig. S5 $F_o - F_c$ omit map of the Zn^{2+} ions in the internal cavity of the cage structure of dimeric cyt *cb*562 (PDB ID: 5AWI). (A) Overall structure of the cage. (B) An enlarged view of the Zn^{2+} ions. The Zn^{2+} ions were omitted from the map calculation. The omit map is shown as blue mesh and contoured at 3.0 σ. The three dimers forming the cage structure are shown in combinations of green and light-green, blue-green and cyan, and red and pink, respectively. The Zn^{2+} ions are shown as green, cyan, magenta, orange, red, yellow, and blue spheres.

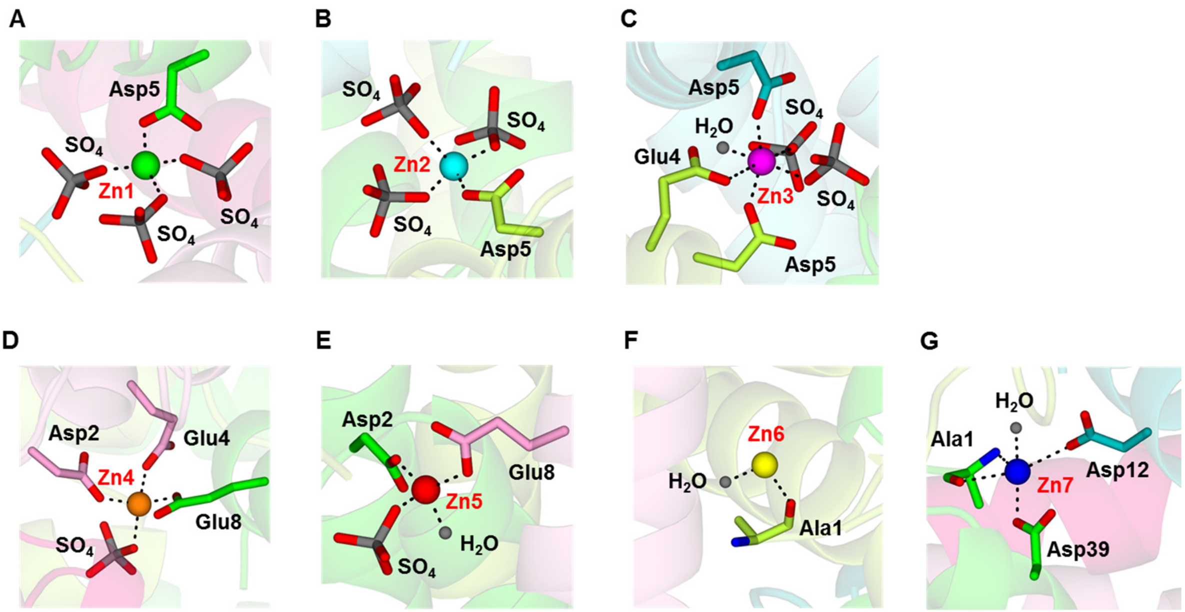

Fig. S6 Coordination structures of twenty one Zn^{2+} and seven SO_4^{2-} ions in the internal cavity of the dimeric cyt *cb*562 cage structure (PDB ID: 5AWI). Fifteen Zn^{2+} ions (Zn1–Zn5) and seven SO_4^{2-} ions form a Zn-SO_4 cluster. The other six Zn^{2+} ions (Zn6 and Zn7) do not participate in the formation of the Zn-SO_4 cluster. (A) The oxygen atoms of three SO_4^{2-} ions (labeled as SO_4) and the side chain of Asp5 of a dimer coordinate to the Zn1 ion. (B) The oxygen atoms of three SO_4^{2-} ions and the side chain of Asp5 of a dimer coordinate to the Zn2 ion. (C) The oxygen atoms of two SO_4^{2-} ions, the side chain of Glu4, the side chains of two Asp5, and one water molecule coordinate to the Zn3 ion. Glu4 and one Asp5 belong to the same dimer, whereas the other Asp5 originates from another dimer. (D) The oxygen atom of one SO_4^{2-} ion and the side chains of Asp2, Glu4, and Glu8 coordinate to the Zn4 ion. Asp2 and Glu4 belong to the same dimer, whereas Glu8 belongs to another dimer. (E) The oxygen atoms of one SO_4^{2-} ion, the side chains of Asp2 and Glu8, and one water molecule coordinate to the Zn5 ion. Asp2 and Glu8 belong to different dimers. (F) The carbonyl oxygen atom of Ala1 and a water molecule coordinate to the Zn6 ion, whereas other water molecules may coordinate to the Zn6 ion. (G) The amino nitrogen and carbonyl oxygen atoms of Ala1, the side chain oxygen atoms of Asp12 and Asp39, and one water molecule coordinate to the Zn7 ion. Ala1 and Asp39 belong to the same dimer, whereas Asp12 belongs to another dimer. The SO_4^{2-} ions, Ala1, and the side chains of Asp2, Glu4, Asp5, Glu8, Asp12, and Asp39 are shown as stick models. The oxygen, nitrogen, and sulfur atoms of the coordinating amino acids are shown in red, blue, and gray, respectively. The coordinating water molecules are shown as small grey spheres. The coordination bonds (< 2.65 Å) between Zn^{2+} ions and their ligands are shown as black dash lines.

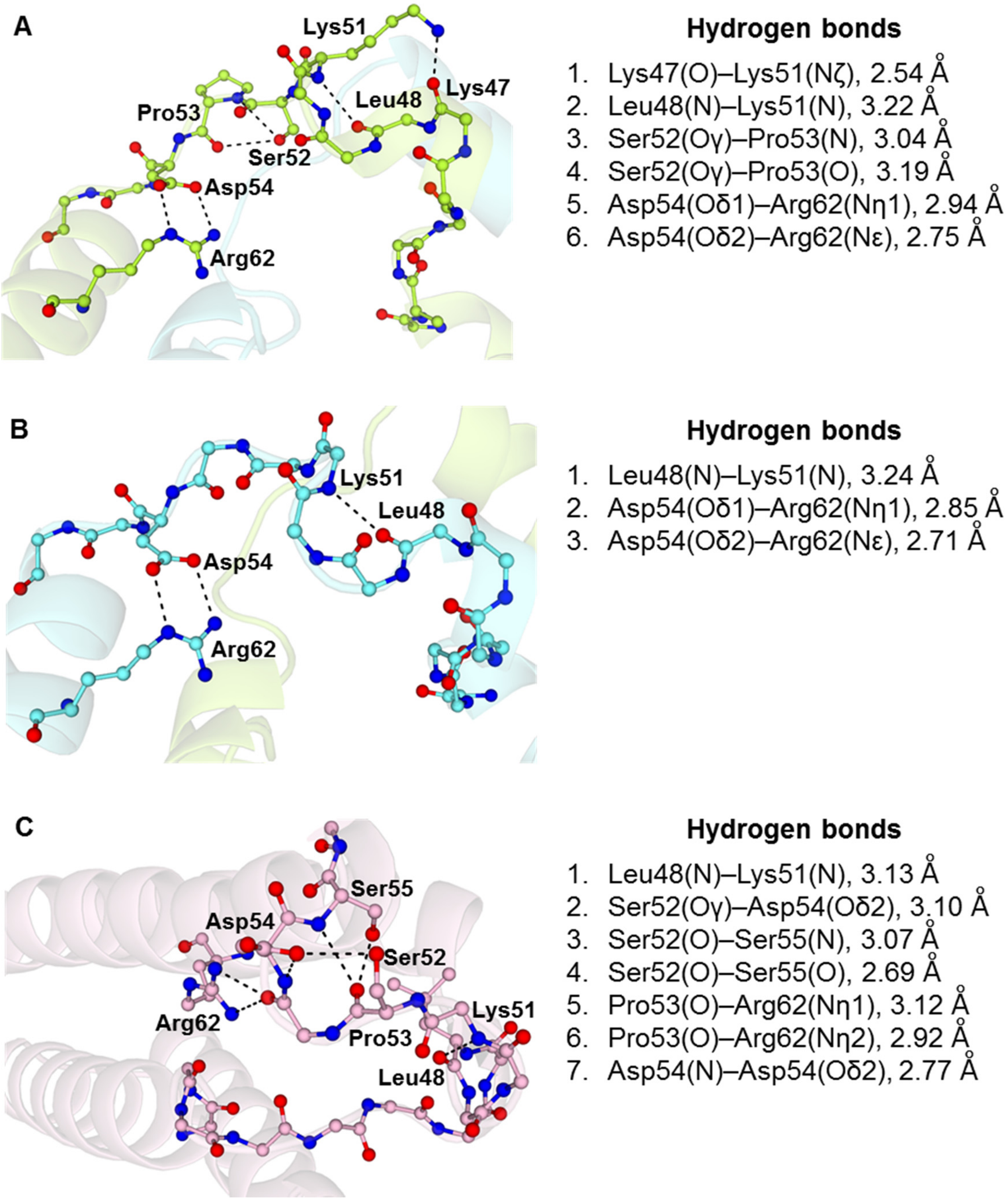

Fig. S7 Schematic views of the hydrogen bonds (< 3.5 Å) at the hinge loop (Lys51–Asp54) of (A, B) the dimeric cyt *cb*$_{562}$ protomers (PDB: 5AWI) and (C) monomeric cyt *b*$_{562}$ (PDB: 256B). The hydrogen bonds are depicted in black dash lines. The cyt *b*$_{562}$ monomer structure was used for the comparison, since the W59 residue of the K59W mutant cyt *cb*$_{562}$ interacted with the hinge loop. The protomers of the dimers are depicted in light-green and cyan, respectively. The main chain of the residues in the loop between helices 2 and 3, and the side chains of the residues forming the hydrogen bonds are shown as ball-and-stick models. The nitrogen and oxygen atoms are depicted in blue and red, respectively.

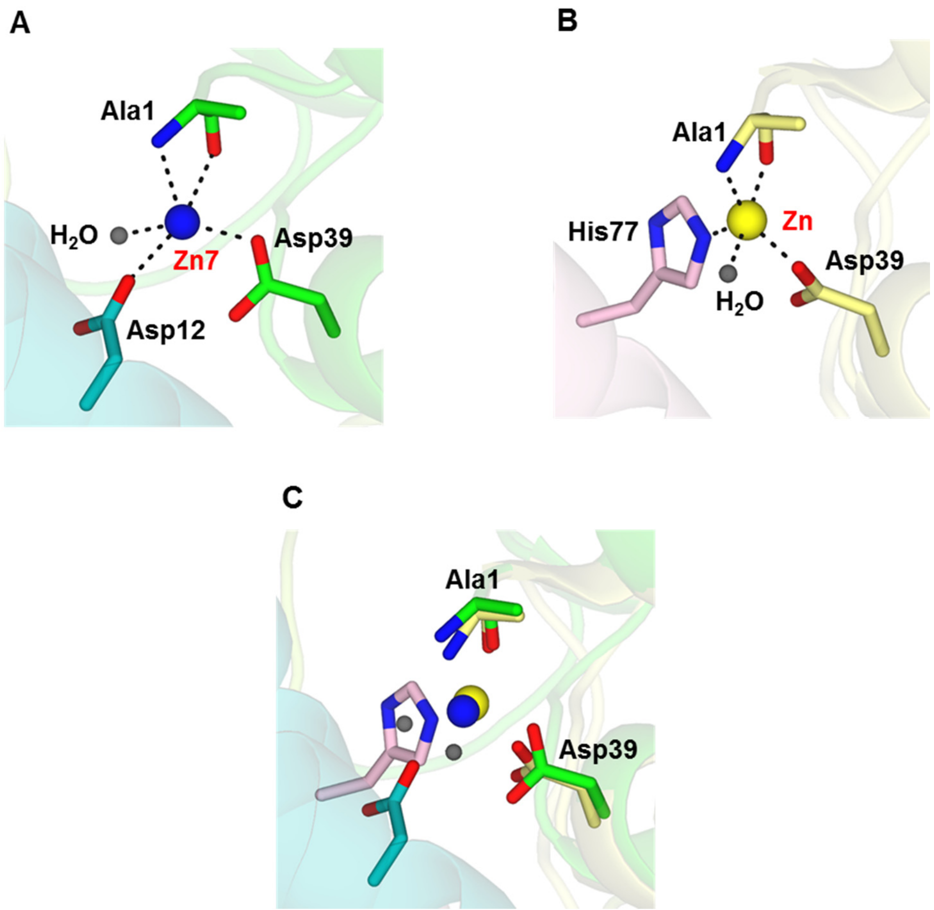

Fig. S8 Schematic views of the Ala1 and Asp39 containing Zn binding sites of (A) domain-swapped cyt cb_{562} dimer cage (PDB ID: 5AWI) and (B) cyt cb_{562} surface mutant cage (PDB ID: 3M4B), and (C) their overlapped view. The coordination bonds (< 2.65 Å) between the Zn^{2+} ions and their ligands are shown as black dash lines. Main and side chains of Ala1 and side chains of Asp12, Asp39, and His77 are shown as stick models. The Zn^{2+} ions are shown as blue and yellow spheres for the domain-swapped cyt cb_{562} dimer cage and cyt cb_{562} surface mutant cage, respectively. The oxygen and nitrogen atoms of the coordinating amino acids are shown in red and blue, respectively. The coordinating water molecules are shown as small grey spheres.

Table S1 Crystallographic statistics of data collection and structure refinement of dimeric cyt cb_{562} (PDB ID: 5AWI).

Data collection	
X-ray source	SPring-8 (BL38B1)
Wavelength (Å)	1.0000
Space group	$P2_13$
Unit cell parameters	
a, *b*, *c* (Å)	94.6, 94.6, 94.6
Resolution (Å)	20.0−1.85 (1.88−1.85)
Number of unique reflections	24682 (1241)
R_{merge}[a]	0.061 (0.594)
Completeness (%)	100.0 (100.0)
$<I/\sigma(I)>$	69.0 (6.2)
Redundancy	16.4 (16.6)
Refinement	
Resolution (Å)	19.3-1.85 (1.90-1.85)
Number of reflections	23154 (1774)
R_{work}[b]	0.180 (0.268)
R_{free}[b]	0.205 (0.391)
Completeness (%)	100.0 (99.9)
Number of atoms in an asymmetric unit	
Protein	1630
Water	61
Heme	86
Average *B* factors (Å^2)	
Protein	37.5
Water	32.4
Heme	25.3
Ramachandran plot (%)	
Favored	99.5
Allowed	0.5
Outlier	0.0

Statistics for the highest-resolution shell are given in parentheses.

[a] $R_{merge}=\Sigma_{hkl} \mid I - <I> \mid (\Sigma_{hkl}\mid I \mid)^{-1}$.

[b] $R_{work}=\Sigma_{hkl} \mid \mid F_{obs} \mid - k\mid F_{calc} \mid \mid (\Sigma_{hkl} \mid F_{obs} \mid)^{-1}$, *k*: scaling factor. R_{free} was computed identically, except where all reflections belong to a test set of 5 % of randomly selected data.

3. Supplementary references

1. J. Faraone-Mennella, F. A. Tezcan, H. B. Gray and J. R. Winkler, *Biochemistry*, 2006, **45**, 10504.
2. E. Arslan, H. Schulz, R. Zufferey, P. Künzler and L. Thöny-Meyer, *Biochem. Biophys. Res. Commun.*, 1998, **251**, 744.
3. E. Itagaki and L. P. Hager, *J. Biol. Chem.*, 1966, **241**, 3687.
4. E. A. Berry and B. L. Trumpower, *Anal. Biochem.*, 1987, **161**, 1.
5. G. Battistuzzi, M. Borsari, M. Sola and F. Francia, *Biochemistry*, 1997, **36**, 16247.
6. M. Yoshida, K. Igarashi, M. Wada, S. Kaneko, N. Suzuki, H. Matsumura, N. Nakamura, H. Ohno and M. Samejima, *Appl. Environ. Microbiol.*, 2005, **71**, 4548.
7. Z. Otwinowski and W. Minor, *Methods Enzymol.*, 1997, **276**, 307.
8. G. N. Murshudov, P. Skubak, A. A. Lebedev, N. S. Pannu, R. A. Steiner, R. A. Nicholls, M. D. Winn, F. Long and A. A. Vagin, *Acta. Crystallogr., Sect. D: Biol. Crystallogr.*, 2011, **67**, 355.
9. G. J. Kleywegt and T. A. Jones, *Acta. Crystallogr., Sect. D: Biol. Crystallogr.*, 1994, **50**, 178.
